# Detection of Sub-acute Brain Injury and Hypoxic-ischemic Encephalopathy using I2C2-WGO and CO-GW-RNN

**DOI:** 10.2174/0115734056352573241118122026

**Published:** 2025-02-21

**Authors:** Priyan Malarvizhi Kumar, Wael Korani, Tayyaba Shahwar, Gokulnath C.

**Affiliations:** 1 Department of Data Science, University of North Texas, Denton, TX, USA; 4 Department of Computing Technologies, SRM Institute of Science and Technology, Chennai, India

**Keywords:** Brain injury, Gadolinium enhancement pattern, Brain MRI, Internal capsule, Recurrent neural network, Wild geese optimization

## Abstract

**Background:**

Hypoxic-ischemic encephalopathy (HIE) is a brain injury that is caused by improper oxygen/blood flow. None of the existing works have concentrated on detecting HIE based on the sub-acute injury in the brain.

**Objective:**

To enhance the accuracy and specificity of HIE detection, a comprehensive approach that includes SAI identification, BGT segmentation, and volume calculation will be utilized.

**Methods:**

This study addresses the critical challenge of detecting hypoxic-schemic encephalopathy (HIE) through advanced image processing techniques applied to brain MRI data. The primary research questions focus on the effectiveness of the proposed CO-GW-RNN method in accurately identifying HIE and the impact of incorporating segmentation and clustering processes on detection performance.

**Results:**

The proposed method achieved remarkable results, demonstrating an accuracy of 98.98% and a specificity of 98.17%, significantly outperforming existing techniques such as the RUB classifier (84.6% accuracy) and the DTL method (94.00% accuracy).

**Conclusion:**

These findings validate the effectiveness of the proposed methodology in improving HIE detection in brain MRI images.

## 1 INTRODUCTION

The brain, which controls the emotions of humans, is a significant and crucial organ in the human body [1, 2]. The deprivation of oxygen and adequate blood flow in the brain leads to HIE and brain damage [3]. The HIE can cause neural impairment and also death in patients if not treated at the starting stage [4]. So, detecting HIE is essential for the patients to undergo proper treatment [5]. To diagnose the HIE, techniques such as ultrasound, MRI, and nucleic acid tests are recommended [6, 7]. However, MRIs with high-resolution images are apt for HIE diagnosis [8]. In brain MRI, key features such as WM, GM, and Cerebro-spinal Fluid (CSF) can be identified with automated processes [9].

In HIE, the WM is affected more than the GM [10]. So, the volume of GM and WM is to be detected for accurate diagnosis. In the existing works, the detection of GM and WM regarding the volume was not done, which could lead to improper HIE detection [11, 12]. Also, in some existing works, the HIE is detected using Deep Neural Network (DNN) and RNN, yet the SAI that might cause mortality if not treated could not be identified by existing models [13, 14]. And the IC, which is the main factor of WM, is to be recognized for the proper diagnosis of HIE [15]. So, by considering the limitations of the existing works, a novel Sbg-WS and CO-GW-RNN-based segmentation of Basal Ganglia and Thalamus (BGT), as well as a diagnosis of HIE, is proposed.

Hypoxic-ischemic encephalopathy (HIE) is a severe brain injury caused by inadequate oxygen and blood flow. Current detection methods often overlook sub-acute injuries, which is crucial for timely intervention. Key brain structures like the Basal Ganglia and Thalamus are often overlooked, and the volume of White Matter and Gray Matter is often overlooked. Deep and Recurrent Neural Networks are used but usually fail to identify sub-acute injuries (SAI) and the internal capsule. A proposed model aims to improve HIE detection by accurately identifying SAI in brain MRI images, using advanced segmentation techniques to isolate affected areas, and measuring the volumes of White Matter and Gray Matter to assess injury severity. The model employs an Iterative Infinite Chaotic map with Collapses Wild Geese Optimization for optimal feature selection and CO-GW-RNN for classification. The model has shown improved accuracy and specificity in HIE detection, providing a more reliable and efficient method for detecting HIE and improving patient outcomes through timely diagnosis.

### 1.1 Problem Statement

The problems observed in the existing works are given below.

(1) The identification of sub-acute brain injury, which is common in infants with mild HIE, is not done in any of the existing works.

(2) In [16-18], the injuries caused in deep GM and the posterior limb of the IC are rarely focused, which leads to worse neuro-developmental outcomes.

(3) The HIE could not be adequately predicted [19-21], as the injury pattern and the respective score group are not considered.

(4) In [6]. did not note the location and extent of ischemic damage involvement (by volume), which affected the HIE prediction.

The contribution of the proposed architecture is expressed by,

(1) The SAI is detected by calculating the Apparent Diffusion Coefficient (ADC) and then finding the injury pattern by GEP.

(2) SS-HC clusters the MRI GM and WM, and then the IC of the WM is calculated.

(3) The BGT region is segmented using Sbg-WS, and the Barkovich Score (BS) technique obtains the score value.

(4) The volume of the GM and WM is found, and the HIE is detected using the CO-GW-RNN classifier.

The remaining paper is arranged as follows: Section 2 discusses the literature survey. Section 3 describes the proposed technique and analyzes its performance in section 4. Lastly, the paper concludes with future work in section 5.

## 2 LITERATURE SURVEY

Midiri *et al*. examined the role of MRI in perinatal hypoxic-ischemic injury and its medico-legal implications. MRI tests were conducted to assess ischemic hypoxic encephalopathy, and data processing was performed accordingly. Features were extracted from the MRI data, and classification was based on hyperexcitability characteristics. Patient characteristics were analyzed, revealing good performance in medico-legal information analysis. However, the use of heterogeneous protocols may lead to missed findings [16].

Piñeiro-Ramos *et al*. examined the metabolic phenotypes of HIE with normal *vs*. pathologic MRI outcomes. Data collection and preprocessing were followed using quantitative gas chromatography-mass spectrometry and untargeted liquid chromatography time-of-flight mass spectrometry for metabolite determination. QC sample analysis was also conducted using two collision energies for metabolite annotation. Results accurately identified normal or pathologic MRI outcomes. However, the inability to collect umbilical cord blood from these patients posed an intrinsic challenge to understanding the metabolic phenotypes of HIE [17].

Banura *et al*. described asymmetry index evaluation of cerebral volume and cerebral blood flow in neonatal HIE using the Rice-Vannucci model. MRI acquisition was conducted initially, followed by preprocessing of the images to assess cerebral volume and cerebral blood flow on both sides affected by HIE. The evaluation time performance was deemed satisfactory; however, the lack of precise control over temperature and duration of hypoxic exposure remained a limitation [18].

Aker *et al*. presented a study on predicting outcomes from MRI and general movements calculation following HIE in low—and middle-income countries based on data from a randomized controlled trial. Initially, data collection from MRI images was conducted, followed by preprocessing. Features were then extracted from the HIE-related data and classified based on MRI data. The results indicated good predictive value for early spontaneous movements and neurodevelopmental outcomes. However, a limitation of the study was the low proportion of infants with severe HIE [19].

Guarnera *et al*. investigated the predictive value of MRI in HIE treated with therapeutic hypothermia. Initially, MRI acquisition was performed, and the data were collected and preprocessed. Statistical analysis was then conducted to measure values, and features were extracted based on MRI data. Subsequently, data classification was performed for HIE. Results indicated high examination values of the apparent diffusion coefficient in the frontal white matter and thalami. However, due to the proven efficacy of therapeutic hypothermia in improving pediatric patients' prognoses, random assignment to therapy was ethically unacceptable [6].

Lee *et al*. examined magnetic resonance spectroscopy of HIE after cardiac arrest. The cardiac arrest protocol was initially utilized to capture magnetic resonance spectroscopy data. Continuous video EEG monitoring signals were collected for preprocessing. Magnetic resonance spectroscopy was conducted to classify data based on neuroimaging structure, followed by statistical analysis of HIE. Results showed higher robustness across cut-off values. However, the failure of oxidative metabolism resulted in severe background voltage suppression in synaptic activity [20].

Boerwinkle *et al*. described the association of network connectivity *via* resting-state functional MRI with consciousness, mortality, and neonatal acute brain injury findings. Initially, data was collected from medical records, categorizing acute-period findings and outcomes utilizing ordinal scores. MRI data acquisition was conducted, and images were classified based on interpreting pediatric neuroradiology. Results indicated a high mean standard deviation of HIE. However, neonatal consciousness and other acute factor outcome measures posed challenges in assessing full-range severity populations [21].

Melanoma, with its high metastatic potential and mortality, is a major challenge in oncology. Early detection reduces skin malignancies like squamous cell carcinoma and melanoma. To address this [22], has introduced an automated diagnostic framework called Multi-scale GC-T2. Utilizing the DermIS and DermQuest datasets, noise reduction, and an Enriched Manta-Ray Optimization Algorithm, this model improves image quality while reducing model complexity. It employs a Multi-scale Graph Convolution Network (M-GCN) for feature extraction, a reward-punishment mechanism, and a sigmoid function for classification. The framework, validated in MATLAB 2020A, performs better than existing models.

Khan *et al*. presents the Dual-3DM3-AD, a multi-modal fusion model for early Alzheimer's Disease (AD) detection. It processes MRI and PET scans using techniques like Quaternion Non-local Means Denoising, Morphology functions, and a Block Divider Model. Key components include a multi-scale feature extraction module, a Densely Connected Feature Aggregator, multi-head attention, and a softmax layer for multi-class diagnosis. The model achieves high performance, with 98% accuracy, 97.8% sensitivity, 97.5% specificity, a 98.2% F-measure, and improved ROC curves [23].

The COVID-19 pandemic has caused significant disruptions in society and the economy, emphasizing the importance of effectively identifying various virus strains. Many current diagnostic techniques are costly and time-consuming, especially in developing nations with inadequate access to health care. Radiological images can be used to analyze pneumonia, one of the virus's main symptoms, and identify its underlying cause. Convolutional Neural Networks (CNNs) are a novel tool for distinguishing between pneumonia, COVID-19-induced pneumonia, and Omicron-variant pneumonia. Even with small datasets, the accuracy of pneumonia detection is improved by using this systematic approach [24].

Epilepsy is a brain disorder causing seizures. Current research has struggled to predict seizures using brain signal data due to issues like overfitting and false positive rates. A deep learning approach called the Deep dual-patch attention mechanism (D2PAM) is proposed to classify pre-ictal signals in patients with epilepsy [25]. This model integrates a deep neural network, reducing patient differences and improving model generalisability and stability. The model outperforms existing models in real patient data, with accuracy rates of 95%, 97%, 99%, 99%, and 99% respectively.

Kujur *et al*. explores the reliance of brain MRI data on CNN-based predictive models for diagnosing Brain Tumor and Alzheimer's Disease. The methodology includes data preprocessing, stratified k-fold cross-validation, and the application of four CNN architectures: S-CNN, ResNet50, InceptionV3, and Xception. To assess their classification performance, these models are tested on two brain MRI datasets, with and without Principal Component Analysis (PCA). The study evaluates and benchmarks the models based on the average Accuracy, Precision, Recall, F1 score, and AUC score derived from stratified five-fold cross-validation. Table **1** shows the performance comparison of the existing techniques [26].

A significant gap exists regarding the volumetric assessment of ischemic damage; previous studies have largely neglected the quantitative analysis of affected brain regions, which is essential for understanding the extent of injury. Finally, while some research has employed machine learning and deep learning techniques, there is a lack of comprehensive approaches that integrate multiple advanced methodologies. The current study aims to fill these gaps by proposing a novel method incorporating BGT segmentation, WM and GM clustering, and SAI detection, resulting in improved classification accuracy (98.98%) and specificity (98.17%) compared to existing techniques. By addressing these identified gaps, this research seeks to contribute to a more nuanced understanding of HIE and its implications for treatment, ultimately aiming to improve patient outcomes through early and accurate detection.

## 3 METHODOLOGY

### 3.1 Proposed Hie Detection Methodology

The proposed model for detecting Hypoxic-Ischemic Encephalopathy (HIE) utilizes Schlieren brightness gradient Watershed Segmentation (Sbg-WS) shown in Fig. (**1**) to segment the Basal Ganglia and Thalamus (BGT) in brain MRI images, allowing for a targeted analysis of the affected regions. It begins by clustering Gray Matter (GM) and White Matter (WM) to distinguish between different brain tissues, followed by identifying the internal capsule (IC), a fundamental structure in motor and sensory processing. The model also detects Sub-Acute Injuries (SAI), which is crucial for determining prognosis and enabling timely treatment. Feature selection uses the Iterative Infinite Chaotic map with Collapses Wild Geese Optimization (I^2^C^2^-WGO) to focus on the most relevant MRI data, enhancing classification accuracy. The final HIE prediction is made with the CO-GW-RNN (COLu-Group Weight-based Recurrent Neural Network) classifier, providing a comprehensive framework for precise HIE detection and improved patient outcomes (Eq **1**).

### 3.2 Input Image

The HIE detection process is initialized by collecting the brain MRI from the patients. The MRI input (*N*) is given by, (Eq. **1**).


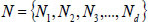
     (1)

Where, (*d*) is the number of input images. Next, (*N*) it is pre-processed, as shown below.

### 3.3 Preprocessing

This section(*N*) is pre-processed to remove unwanted artifacts and enhance the image for accurate processing. The preprocessing steps are given by,

#### 3.3.1 Normalization

Here, the Z-Score technique uses the mean and standard deviation of (*N*) is to normalize the input image. It is equated as, (Eqs. **2**-**4**).


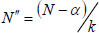
     (2)


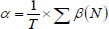
     (3)


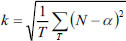
     (4)

Where, (*N"*) is the normalized image, (*α*) is the mean, (*β*) is the pixel intensity of (*N*), (*T*) is the total number of pixels in the input image, and (*k*) is the standard deviation of (*N*).

#### 3.3.2 Denoising

After normalizing, the salt and pepper noise in (*N"*) is removed using an Anisotropic Diffusion Filter (ADF) that updates the pixel values based on the gradient of the image as, (Eq. **5**).


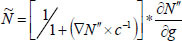
     (5)

Where (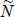) the denoised image (▼) is, the gradient between the pixels of the input image (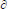) is the differential operator, (*c*) and the constant value (*g*) is the iteration.

#### 3.3.3 Contrast Enhancement

Next, the contrast (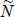) is enhanced using Contrast-Limited Adaptive Histogram Equalization (CLAHE), which considers every pixel to increase the contrast. CLAHE is a strongly non-linear algorithm that enhances image contrast, particularly in medical imaging like MRI, where its application can significantly impact diagnostic information in brain images. CLAHE improves the visibility of structures by enhancing contrast in localized regions, making subtle abnormalities and lesions more discernible, which aids radiologists in diagnosing conditions such as hypoxic-ischemic encephalopathy (HIE). However, while enhancing contrast, CLAHE can also amplify noise in areas with low signals, potentially leading to misinterpretations of noise as pathological findings; thus, careful application and parameter tuning are essential to mitigate this risk. Additionally, CLAHE addresses non-uniform illumination issues common in MRI images by normalizing intensity across the image, facilitating better assessment of brain structures and lesions. However, the algorithm's non-linear nature may introduce variability in quantitative analyses, affecting the accuracy of measurements, such as volume assessments of specific brain regions, and potentially leading to misinterpretations of injury or disease extent. Furthermore, CLAHE's application may result in subjective variations in image interpretation among different clinicians, with some finding enhanced images easier to read while others prefer the original images. Therefore, while CLAHE can enhance diagnostic information, its risks related to noise amplification and variability in assessments necessitate careful consideration and validation within specific diagnostic contexts. The histogram (*x*) of (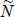) is initially equated with the number of pixels (*G*) and the number of grey scales 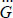 in (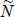) as, (Eq. **6**).


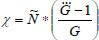
     (6)

Next, the input image's over-amplification is reduced by clipping, and then the image is normalized to make the contrast equal throughout the input, as follows (Eqs. **7**, **8**).


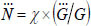
     (7)


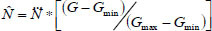
     (8)

(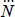) the clipped image (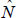) is the contrast-enhanced image, and (*G_max_*, *G_min_*) the input image's maximum and minimum pixel values are. Next, the clustering of the GM and WM i(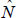)s done.

### 3.4 Clustering

Here, the (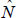) SS-HC technique clusters the GM, WM, and CSF. The Hierarchical Clustering (HC) that does not need the number of clusters beforehand and thus helps when the data does not lend to a particular number of clusters is used. On the contrary, the choice of distance metrics can affect the clustering result. Therefore, the Sneath and Sokal (SS) distance is used to overcome the suboptimal solution instead of the traditional distance measure of HC. The SS-HC technique is explained below.

Let the pixels (*D*) with the (*f*) number of pixels in (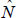) be given by, (Eq. **9**).


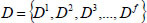
     (9)

Now, each pixel is considered its cluster, so the GM, CSF, and WM can be clustered effectively. Next, the distance (*ε*) between the clusters is identified using the SS distance formula, which calculates the distance based on the pixel values, (Eq. **10**).


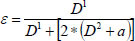
     (10)

Where, (*a*) is the constant value. Then, the closest cluster is merged regarding the minimum distance as, (Eqs. **11**, **12**).


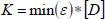
     (11)


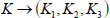
     (12)

(*K*) the clustered output (*K_1_*) is the GM, (*K_2_*) the WM, and (*K_3_*) the CSF. The clustering is repeated after merging the clusters into a single form until all the data points are clustered (*ε*). Next, the IC in the WM is identified as shown below.

### 3.5 Internal Capsule Identification and Volume Calculation

The IC is a WM structure that connects the cerebral hemisphere to the thalamus, brain stem, and subcortical structures. Thus, the brighter pixels (*K_2_*) are the IC and are represented as (*B*). Now, from (*B*) and (*K_2_*), the volume calculation is done as described below (Eqs. **13**, **14**).


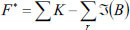
     (13)


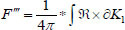
     (14)

Where, (F*) is the volume of WM, (*r*) is the number of pixels in (*B*), (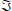) is the probability function of the pixel intensity in (*B*), (*F''''*) is the volume of GM, (*π*=22/7)(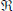) is the pixel intensity in (*K_1_*), and 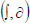 are the integral and differential operators. Now, the total volume (*E*) is equated by, (Eq. **15**).


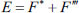
     (15)

Meanwhile, from the pre-processed image, the SAI of the brain is identified as described in Section 3.5.

### 3.6 Sub-acute Injury Detection

From the pre-processed output (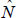), the SAI that causes the bleeding to slower and become life-threatening if untreated is detected using ADC value and GEP as,

#### 3.6.1 ADC value

Initially, the ADC, which determines the water molecules in the tissue regarding (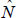), is equated by, (Eq. **16**).


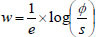
     (16)

Where, (*w*) is the ADC value, (*e*) is the constant, (Ø) is the pixwl intensity of (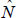), and (*s*) is the number of pixels in (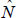).

#### 3.6.2 Pattern Calculation

Now, the pattern i(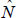)s identified to find the SAI using GEP, which enhances certain tissues and lesions, making them more conspicuous and making it easier to detect SAI on MRI. The SAI detected the image (*H*) is thus evaluated by, (Eq. **17**).


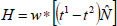
     (17)

where (*t^1^*, *t^2^*) are the longitudinal and latitudinal enhancement values (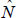). At the same time, the segmentation (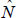) is done as explained below.

### 3.7 Segmentation and Score Calculation

Meanwhile, (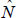) the BGT area is segmented using the Sbg-WS technique. Here, the Watershed Segmentation (WS), which depends on the intensity gradients of the input image, is used for BGT segmentation. Yet, the region with low contrast could not be adequately segmented, which reduces the segmentation accuracy. So, the Schlieren brightness gradient (Sbg) is used to brighten the input image, and then the segmentation is carried out. The Sbg-WS process is expressed by,

The brightness is increased in each direction (*n*, *o*, *p*)(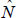) using the Sbg method, which improves the contrast regarding the pixel density (*γ*) in each direction. The brightness improved the image (*V*) is thus given by, (Eq. **18**).


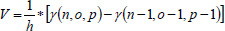
     (18)

Where, (*h*) is the coefficient parameter, and *γ*(*n*-1, *o*-1, *p*-1) are the neighbouring density thickness. Next, the influence zone (*A*), which is the catchment area, is equated by, (Eq. **19**).


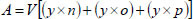
     (19)

Where (*y*) is the intensity gradient of (*V*). Now, the catchment basin (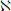) that helps in detecting the BGT area is calculated as, (Eq. **20**).


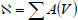
     (20)

Finally, the image is mapped to the (*C*)(*V*) Boundary of the segment of the BGT in an MRI. It is expressed as, (Eq. **21**).


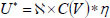
     (21)

where (U*) is the BGT segmented image and (*η*) the mapping function of (*V*). The pseudo-code for Sbg-WA is given below.


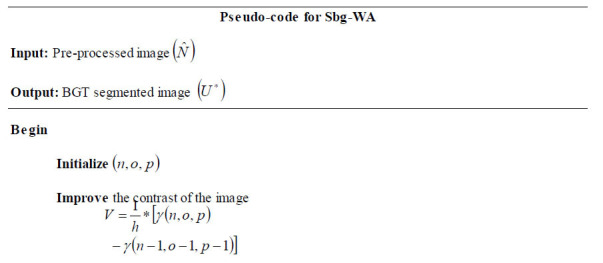



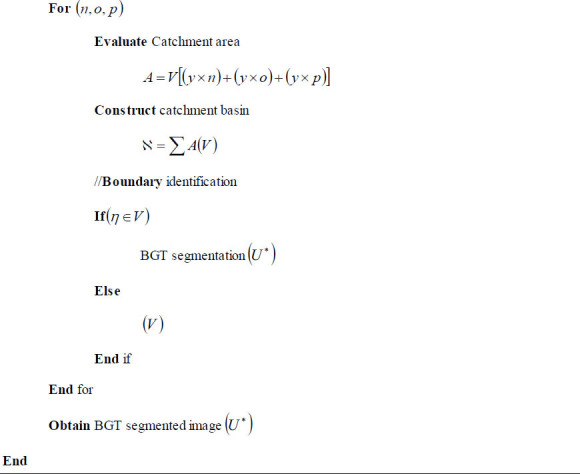


Next, the Barkovich score (*U*), which plays an essential factor in identifying the HIE, is calculated regarding (U*), (Eq. **22**).


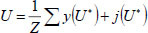
     (22)

where (*Z*) is the number of pixels in (U*), (*y*) is the pixel intensity gradient, and (*j*) is the volume of (U*) regarding (*y*). Then, the features are extracted from (*U*) the volume output and the SAI-detected image is used for further processing.

### 3.8 Feature Extraction

Now, from Barkovich's score (*U*), volume calculation output (*E*), and SAI-identified image (*H*), features such as the Gray Level Run Length Matrix (GLRLM), Local Binary Pattern (LBP), Gray Level Co-occurrence Matrix (GLCM), Histogram of Oriented Gradient (HOG), Laplacian of Gaussian (LoG), Rotation Invariant Local Binary Patterns (RILBP), intensity, shape, and texture features are extracted.

The study involved extracting multiple texture features to improve the analysis of brain MRI images to identify hypoxic-ischemic encephalopathy (HIE). One of the main features is the Gray Level Run Length Matrix (GLRLM), which calculates the distance between consecutive pixels of the same intensity. This information can be used to identify patterns that may indicate pathological changes or to gain insights into texture uniformity. The Local Binary Pattern (LBP) evaluates the local image structure by comparing each pixel with its neighbours. This technique is crucial for medical imaging because it captures micro-patterns and is resilient to changes in illumination. To describe the textures of brain tissue and identify anomalies, the Gray Level Co-occurrence Matrix (GLCM) assesses the spatial relationships between pixels. It produces statistical measures like contrast, correlation, energy, and homogeneity. The Histogram of Oriented Gradient (HOG) analyses the distribution of gradient orientations, which helps find the edges and shapes necessary for locating lesions or structural alterations. For edge detection, the Laplacian of Gaussian (LoG) is employed to highlight regions of unexpected intensity change, essential for evaluating brain damage. While general Intensity, Shape, and Texture Features cover a variety of metrics that characterize the general properties of brain tissue, Rotation Invariant Local Binary Patterns (RILBP) offer consistent texture analysis independent of orientation. These features were selected because they are sensitive to small variations in tissue texture that could indicate ischemic damage, resilient to changes in noise and lighting, and can provide a thorough analysis that improves the precision of HIE detection and prediction. The ultimate objective of obtaining these texture features is to enhance diagnostic capacities by providing an in-depth understanding of the structure and texture of brain tissues, which are essential for recognizing and evaluating HIE.

It is represented by, (Eq. **23**).


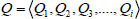
     (23)

where (*Q*) is the extracted feature and (*i*) is the number of (*Q*). After removing the features, the optimal features are selected using the I^2^C^2^-WGO method.

### 3.9 Feature Selection

In this phase, the optimal features are selected (*Q*) using I^2^C^2^-WGO. Here, the Wild Geese Optimization (WGO), which exhibits fast convergence rates and is computationally efficient for selection in search space, is used to find the essential features for HIE prediction. However, the search in the exploration phase might be difficult due to the deceptive local optima problem. So, premature convergence is avoided by using an Iterative Infinite Chaotic map with Collapse (I^2^C^2^) in the exploration phase. The I^2^C^2^-WGO method is detailed below.

#### 3.9.1 Initialization

The population (*Q*) of Wild Geese (WG), which is the extracted feature and the initial position (*n_b, v_*) for (*b^th^*) WG in (*v^th^*) search space, are initialized as, (Eqs. **24**, **25**).


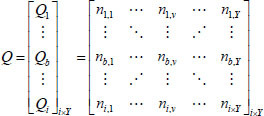
     (24)


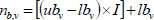
     (25)

Where, (*Q_b_*) is the (*b^th^*) WG, (*i*) is the number of WG with search dimension (*Y*), (*lb_v_*, *ub_v_*) are the lower and upper bound values in (*v^th^*) search space, and (*I*) is the random value. Next, the fitness function (*λ*) evaluated as,

#### 3.9.2 Fitness

Here, it (*λ*) is calculated based on the maximum classification accuracy (*P*) and is given by, (Eq. **26**).


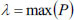
     (26)

Where, (*P*) is the classification accuracy. The position of WG (*λ*) is updated to find the optimal solution.

#### 3.9.3 Position Update

The WG migrates to the new position and then searches for food in the search space. The proposed optimizer's position update is as follows.

#### 3.9.4 Migration

Here, the WG migrates as a whole based on the velocity and displacement factor (*K_1_*, *K_2_*) of WG. Here, the migration area is selected based on I^2^C^2^, which chooses the migration area regarding the displacement of WG. It is equated by, (Eqs. **27**, **28**).


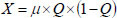
     (27)


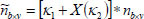
     (28)

Where (*X*) is the migration area, (*μ*) is the mapping function, and 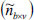 is the new position of WG.

#### 3.9.5 Search for Food

Next, the WG searches for food regarding 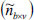
along the food direction and is given by, (Eq. **29**).


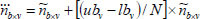
     (29)

Where, 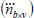 is the new position of WG, and (*N*) is the iteration. Thus, WG's position gets updated regarding (*λ*) WG's death and, again, migration. The final updated position, which is the optimal feature (*S*), is represented as, (Eq. **30**).


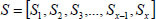
     (30)

where (*S*) is the selected feature and (*x*) is the number of (*S*). Next, the HIE in input MRI is predicted using (*S*) method below.

### 3.10 HIE Prediction

Here, the presence of HIE is detected by (*S*) using CO-GW-RNN. As the previous memory is maintained through recurrent connections, the contextual patterns are identified effectively by RNN. However, each piece of data is processed separately, which increases the processing time. So, to overcome this issue, the classifier's input is regularized using Group Weight (GW) regularization and activated by the COLu (CO) activation function. The COLu (CO) activation function, utilized in the CO-GW-RNN (COLu-Group Weight-based Recurrent Neural Network) architecture for Hypoxic-Ischemic Encephalopathy (HIE) detection, plays a vital role in enhancing the model's ability to learn complex patterns from MRI data. By introducing non-linearity, the COLu function enables the network to capture intricate relationships indicative of HIE while facilitating adequate gradient flow during training, which is essential for deep learning. Its potential regularization properties may also help prevent overfitting, which is particularly important in medical datasets with limited samples. The architecture of CO-GW-RNN is given in Fig. (**2**).

The process of the CO-GW-RNN classifier is explained in detail below.

#### 3.10.1 Regularization

Initially, the GW regularization that adds the weight value to capture the critical information is used to parallelize the process for accurate HIE prediction. It is expressed as, (Eq. **31**).


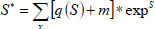
     (31)

Where, (S*) is the regularized output, (*q*, *m*) is the weight and bias value, and (exp) is the exponential factor. Next, (S*) it is given as input to the hidden layer.

#### 3.10.2 Hidden Layer

Here, the data from the previous output is collected, and along with (S*) it, the data is activated by the CO activation function (Ω), which reduces the processing time using the inverse processing of the input data. The hidden layer output (*R^u^*), with time (*u*) is equated as, (Eqs. **32**, **33**)


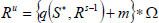
     (32)


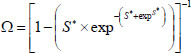
     (33)

where (*R^u-1^*) is the production of the previously hidden layer concerning the time(*u*-1). Next, (*R^u^*) is given to the output layer to get the classified results.

#### 3.10.3 Output Layer

The final classified output (*J*) is evaluated regarding the present hidden state output (*R^u^*), and (Ω) as, (Eqs. **34**, **35**).


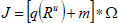
     (34)


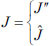
     (35)

Where (*J"*) represents the normal class and (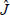) is the HIE class. The pseudo-code for CO-GW-RNN is given below.


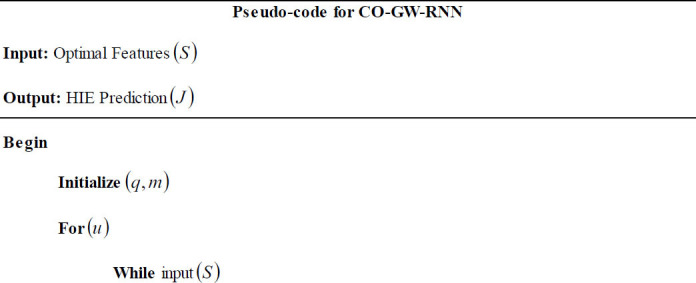



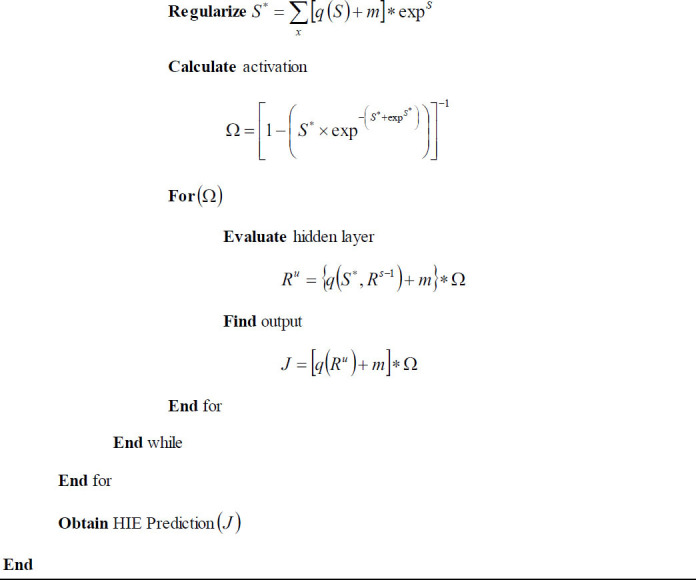


Thus, the SAI is identified, BGT is segmented and the HIE in the brain MRI is predicted effectively. The performance evaluation of the proposed framework is described in Section 4.

## 4 RESULTS AND DISCUSSIONS

This section analyzes the performance of the proposed sub-acute brain injury detection framework in terms of various metrics. The proposed work is implemented in the PYTHON platform for the performance evaluation, which has wider data interpretability.

### 4.1 Dataset Description

The performance evaluation of the proposed system utilizes the Boston Neonatal Brain Injury dataset, which includes raw diffusion-weighted MRI images, derived diffusion parameter maps, and manually annotated lesion marks from 133 patients. For the evaluation, the dataset is split into 80% for training and 20% for testing, ensuring that the model is adequately trained on a substantial amount of data to enhance its learning and generalization capabilities. The results of the proposed approach are presented in Table **2**, which includes key performance metrics such as accuracy, sensitivity, and specificity, along with visual examples of lesion segmentation or classification. This table compares the proposed system's performance against existing methods. Overall, the evaluation demonstrates the effectiveness of the proposed approach and highlights its potential for clinical application in diagnosing and monitoring brain injuries in neonates.

Table **2** illustrates the image outcome of input, noise removed, skull stripped, contrast-enhanced, pattern enhanced, clustered CSF, GM, and WM, and segmented brain MRI of normal and HIE.

### 4.2 Implementation Platform

The framework designed for detecting Hypoxic-Ischemic Encephalopathy (HIE) leverages the capabilities of the Python programming language, which is highly regarded for its flexibility and a wide range of libraries tailored for data analysis and machine learning. Essential libraries such as TensorFlow and PyTorch support deep learning applications, while NumPy facilitates numerical computations. For data visualization, tools like Matplotlib and Seaborn are invaluable. This rich ecosystem allows for developing and training sophisticated models, such as the CO-GW-RNN (COLu-Group Weight-based Recurrent Neural Network) employed in this study. By implementing the framework in Python, we can efficiently handle large datasets and seamlessly integrate various preprocessing techniques, ultimately enabling rigorous performance evaluations through metrics like accuracy, precision, recall, and F1-score.

### 4.3 Performance Analysis

The proposed CO-GW-RNN's performance for classifying HIE is primarily assessed by weighing against the prevailing techniques, namely RNN, Deep Neural Network (DNN), Convolutional Neural Network (CNN), and Deep Belief Network (DBN).

Fig. (**3**) illustrates the performance of CO-GW-RNN in terms of accuracy, precision, recall, and Mathew's Correlation Coefficient (MCC). The proposed method attained 98.95% and 98.39% accuracy and precision, respectively. Meanwhile, the existing RNN attained a lower accuracy of 96.38%, and CNN achieved a recall of 90.61%. This is due to the non-concentrated restricted learning performance of the existing techniques. Also, Table **3** depicts the performance of the proposed method regarding F-measure, specificity, True Positive Rate (TPR), Positive Predictive Value (PPV), and False Negative Rate (FNR). The proposed approach achieves an improved specificity of 98.17%, PPV of 98.39%, and FNR of 0.003. However, the existing methods achieved an average of 91.39% specificity and 90.11% PPV, which are lower than the proposed method. As significant information is captured through GW regularization by adding weights to the network, the proposed method classifies HIE as having higher performance than the existing methods.

Fig. (**4**) shows an evaluation result of the I2C2-WGO, in which the fitness performance of the proposed I2C2-WGO algorithm demonstrates a clear advantage over existing algorithms such as Wild Goose Optimization (WGO), Osprey Optimization Algorithm (OOA), Butterfly Optimization Algorithm (BOA), and Walrus Optimization Algorithm (WaOA). I2C2-WGO consistently achieved an average fitness of 95.05% across multiple iterations, surpassing the other algorithms. A key factor in this success is the algorithm's ability to address premature convergence during exploration, a common issue where optimization algorithms settle into local optima too early, limiting performance. The I2C2 mechanism effectively mitigates this by mapping the migration area of the Wild Goose more efficiently, enhancing exploration and allowing I2C2-WGO to find better solutions. In contrast, OOA and BOA, which suffer from premature convergence, achieved lower average fitness scores of 89.42% and 87.71%, respectively. This limitation in existing algorithms restricts their ability to search the solution space as effectively as I2C2-WGO. Thus, the proposed algorithm consistently outperforms traditional methods, maintaining superior fitness throughout optimization.

The performance of BGT segmentation using the proposed method is analyzed in Fig. (**5**) regarding the Silhouette Score (SS) and Jaccard Index (JI) by comparing with prevailing methods, namely WS, Otsu Thresholding (OT), Active Contour (AC), and Canny Edge Detector (CED). The proposed method achieved 0.95 SS and 0.96 JI, higher than the existing approaches. Meanwhile, the existing WS attained 0.91 JI, OT attained 0.89 SS, and AC attained 0.84 JI. As the low contrast regions are effectively segmented through the Sbg technique, the proposed method segmented BGT with increased SS and JI. Thus, the segmentation performance of Sbg-WS is enhanced over the prevailing methods.

Fig. (**6**) presents a performance analysis of the SS-HC (Spectral Similarity-based Hierarchical Clustering) algorithm in clustering white matter (WM) and gray matter (GM) of the brain, demonstrating its efficiency compared to established algorithms like Hierarchical Clustering (HC), Farthest First Clustering (FFC), K-means, and Density-Based Spatial Clustering of Applications with Noise (DBSCAN). SS-HC completed the clustering in just 3654 milliseconds, significantly faster than FFC (6891 ms) and DBSCAN (8128 ms). This efficiency is attributed to its effective distance evaluation using the SS measure, which minimizes sub-optimal clustering and enhances accuracy. Overall, SS-HC’s combination of rapid processing and improved clustering quality positions it as a superior tool for neuroimaging applications, facilitating timely and precise analysis of brain structures.

Figs. (**7a** and **7b**) represent the performance of the SS-HC method regarding Dice Score (DS) and Variance Ratio (VR). From Fig. (**7a**), the proposed SS-HC attained a higher DS of 0.95; the existing HC attained 0.91 DS, and K-means attained 0.84 DS. The proposed SS-HC achieves optimal clustering with better performance as the WM and GM regions are clustered based on the SS distance measure. Moreover, as in Fig. (**7b**), the higher VR of 0.95 is attained by the SS-HC algorithm. However, the existing methods attained an average VR of 0.86, lower than the proposed algorithm. Hence, the WM and GM of brain MRI are more accurately clustered using the proposed algorithm.

### 4.4 Comparative Analysis

The proposed work's enhanced performance is verified by comparing it with related works of HIE classification in terms of accuracy and specificity.

Table **4** and Fig. (**8**) show the comparison of the proposed model with existing technology, such as the Random Undersampling Boosting (RUB) classifier, Deep Transfer Learning (DTL), Residual Neural Network18 (ResNet18), Random Forest (RF), and Gradient Boosting (GB). The RUB classifier achieved 84.6% accuracy, RF reached 95% accuracy, DTL demonstrated 88% specificity, and ResNet18 recorded 72.15% accuracy. However, these methods do not incorporate critical processes such as Basal Ganglia and Thalamus (BGT) segmentation, White Matter (WM) and Gray Matter (GM) clustering, or volume calculation, which limits their overall performance. In contrast, the proposed method addresses these limitations by including BGT segmentation, WM-GM clustering, and volume calculation, leading to a more accurate and comprehensive analysis of Hypoxic-Ischemic Encephalopathy (HIE). As a result, it achieves a significantly higher accuracy of 98.98% and a specificity of 98.17%. By focusing on these additional processes, the proposed approach enhances performance and offers a clear improvement over the existing techniques.

## CONCLUSION

This study presents a novel approach for the detection of Hypoxic-Ischemic Encephalopathy (HIE) through the integration of Sbg-WS (Segmentation-based Weight Selection) and CO-GW-RNN (COLu-Group Weight-based Recurrent Neural Network) methodologies. The proposed framework effectively segments critical brain structures, such as the Basal Ganglia and Thalamus, and accurately predicts the presence of HIE from MRI scans. Performance evaluation on the Boston Neonatal Brain Injury dataset demonstrates that the CO-GW-RNN model outperforms existing techniques, achieving high F-Measure, specificity, true positive rate, and positive predictive value. The results indicate that this model is a promising tool for enhancing the diagnosis of HIE, which is crucial for timely intervention and treatment. This research addresses the limitations of previous methods and contributes to ongoing efforts to improve neonatal care through advanced imaging techniques.

## LIMITATIONS AND FUTURE WORK

The proposed model for detecting Hypoxic-Ischemic Encephalopathy (HIE) has several limitations, including a small and potentially non-diverse dataset, challenges in segmenting low-contrast regions, a narrow focus on sub-acute injuries, and ethical concerns around data collection from vulnerable populations. In future work, Heart Rate Variability (HRV) will be focused on differentiating the mild and moderate/severe forms of HIE.

## Figures and Tables

**Fig. (1):**
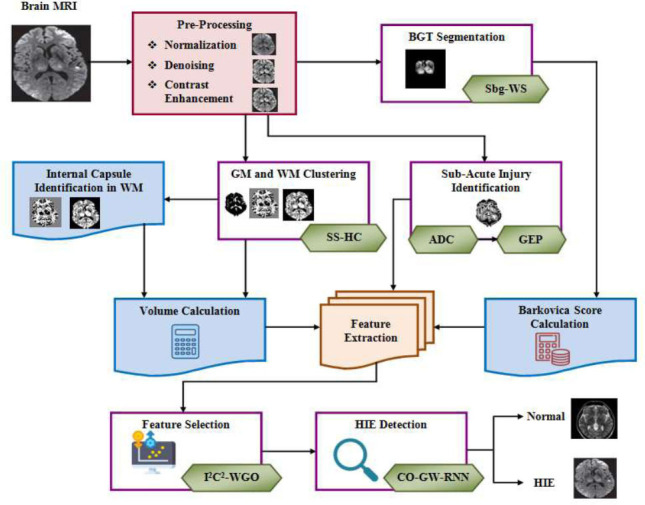
Architecture of the proposed framework.

**Fig. (2):**
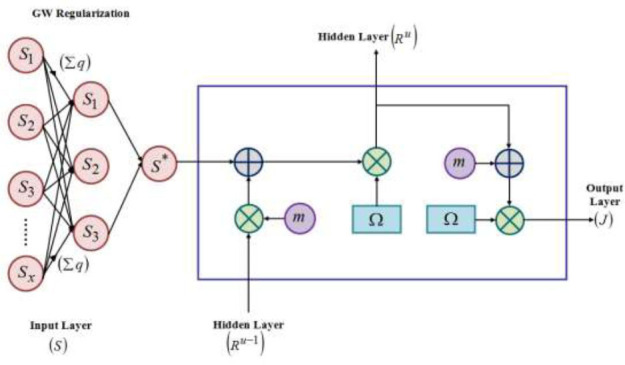
CO-GW-RNN classifier.

**Fig. (3):**
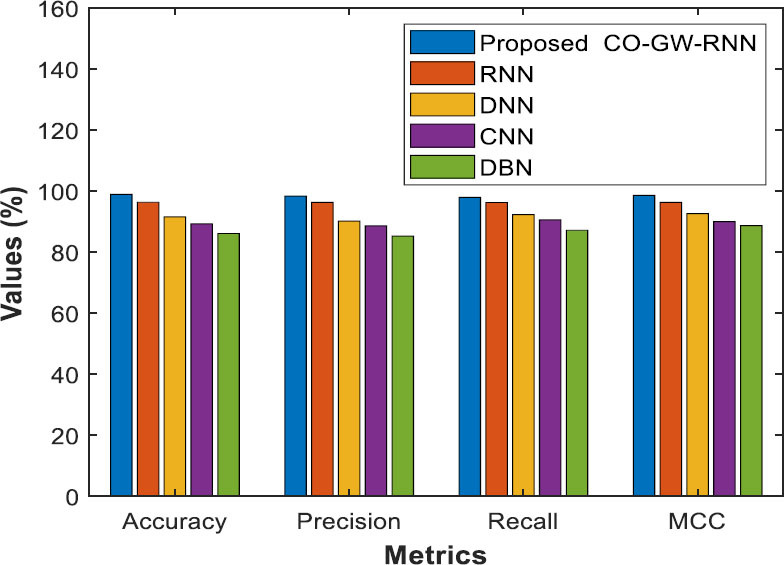
Performance comparison of CO-GW-RNN.

**Fig. (4):**
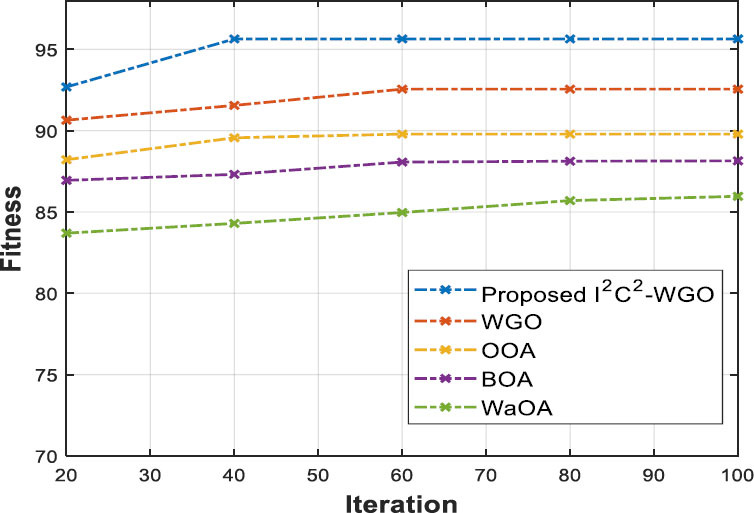
Evaluation of I^2^C^2^-WGO.

**Fig. (5):**
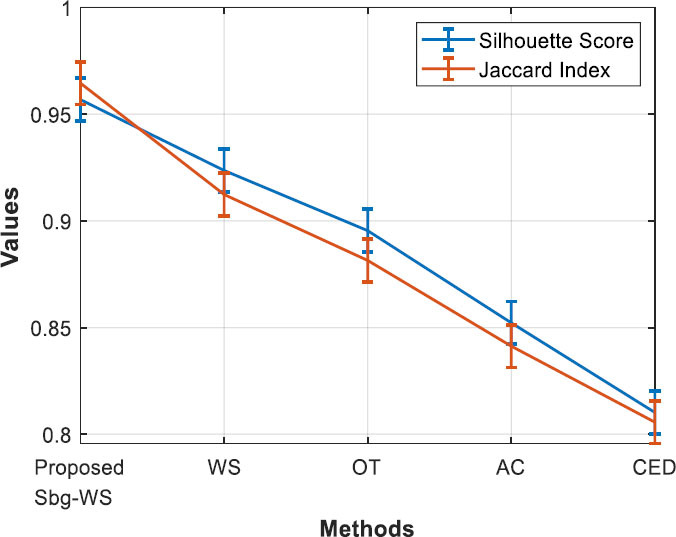
Segmentation analysis of Sbg-WS.

**Fig. (6):**
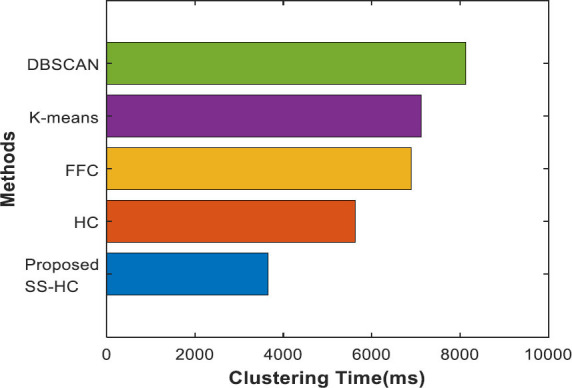
Analysis of proposed SS-HC.

**Fig. (7a):**
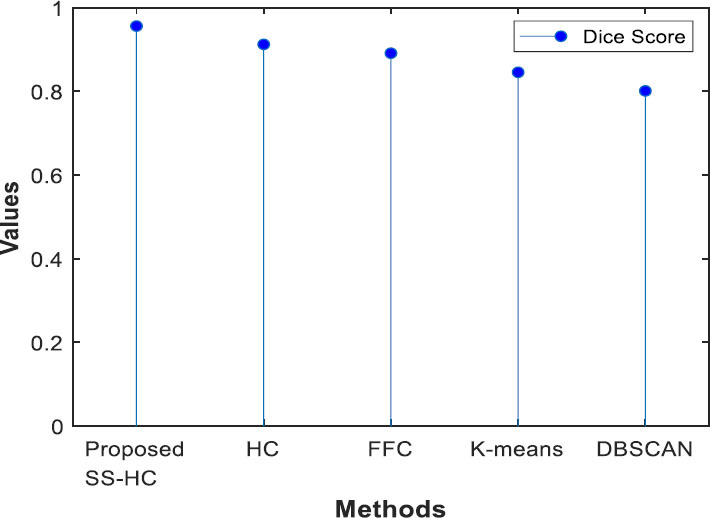
Dice score analysis of proposed SS-HC.

**Fig. (7b):**
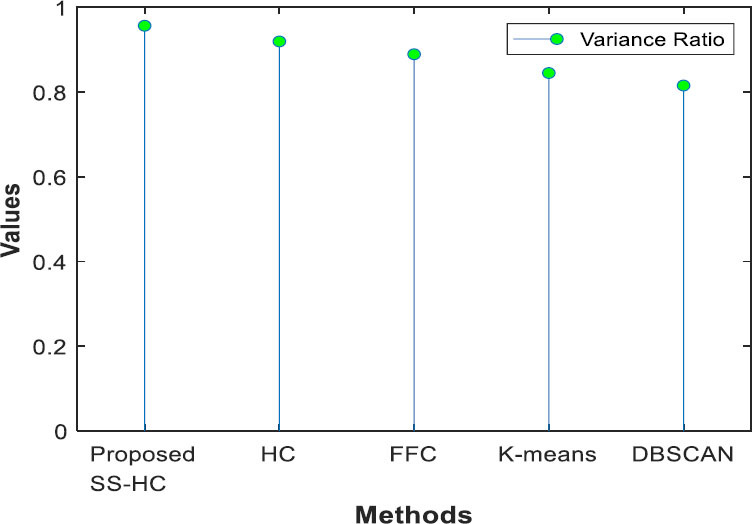
Variance ratio evaluation.

**Fig. (8):**
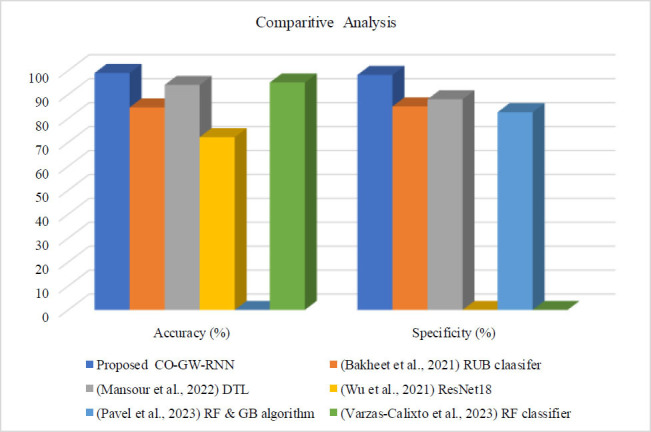
Comparative analysis of the proposed and the existing techniques.

**Table 1:** Performance comparison of existing techniques.

**Reference**	**Methodology**	**Results**	**Limitations**
[6]	MRI data acquisition and statistical analysis for HIE classification.	High predictive value for early spontaneous movements and neurodevelopmental outcomes.	Low proportion of infants with severe HIE in the study.
[20]	Magnetic resonance spectroscopy post-cardiac arrest with continuous EEG monitoring.	High robustness across cut-off values for HIE classification.	Severe background voltage suppression in synaptic activity due to oxidative metabolism failure.
[21]	Resting-state functional MRI to assess network connectivity and outcomes.	The high mean and standard deviation of HIE findings.	Challenges in assessing full-range severity populations and neonatal consciousness.
[9]	Development of a clinical risk scoring system for neurodevelopmental outcomes.	Novel scoring system for predicting outcomes in HIE survivors.	Limited validation across diverse populations and settings.
[5]	Color Doppler ultrasonography to assess early brain injury in infants.	An association was found between early perfusion and brain injury.	Focused primarily on early detection, lacking comprehensive analysis of long-term outcomes.
[1]	A systematic review of HIE codes for resource-limited settings.	Identified key factors for HIE management in low-resource environments.	The findings are limited to high-resource settings and may not address all clinical scenarios.

**Table 2:** Image results.

**Classes**	**Normal**	**HIE**
**Input image**	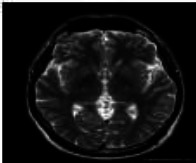	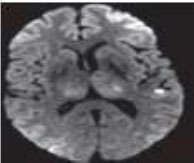
**Noise removed image**	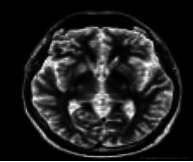	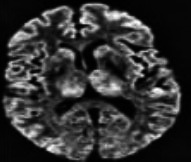
**Skull stripped image**	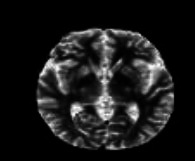	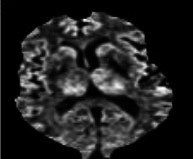
**Contrast-enhanced image**	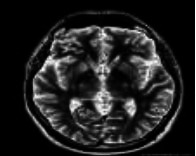	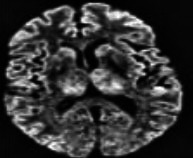
**Pattern enhanced image**	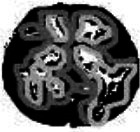	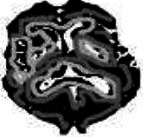
**Cerebro Spinal Fluid (CSF) image**	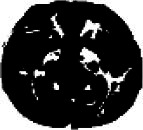	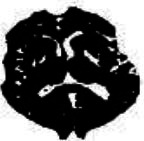
**WM image**	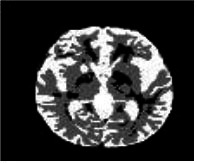	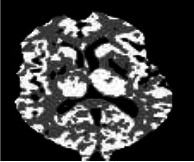
**GM image**	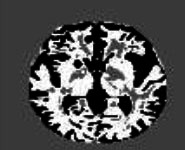	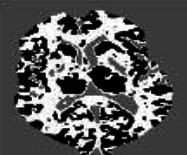
**Segmented image**	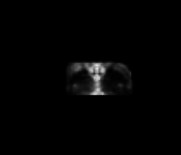	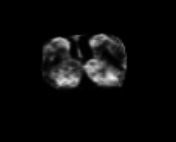

**Table 3:** Analysis of proposed CO-GW-RNN.

**Techniques**	**F-Measure (%)**	**Specificity** **(%)**	**TPR** **(%)**	**PPV (%)**	**FNR (%)**
Proposed CO-GW-RNN	98.124	98.174	98.012	98.397	0.0035
RNN	96.874	96.899	96.312	96.314	0.0078
DNN	92.389	91.344	92.310	90.234	0.0096
CNN	90.062	90.601	90.617	88.612	0.0120
DBN	88.012	86.745	87.214	85.314	0.0198

**Table 4:** Comparative analysis.

**References**	**Technique used**	**Accuracy (%)**	**Specificity (%)**
Proposed	CO-GW-RNN	98.98	98.17
[27]	RUB classifier	84.6	85
[28]	DTL	94.00	88.00
[29]	ResNet18	72.15	-
[30]	RF & GB algorithm	-	82.6
[31]	RF classifier	95	-

## Data Availability

The data and supportive information are available within the article.

## LIST OF ABBREVIATIONS

HIE: Hypoxic-ischemic encephalopathy
SAI: Identify Sub-acute Injuries
CSF: Cerebro-spinal Fluid
BGT: Basal Ganglia and Thalamus
ADC: Apparent Diffusion Coefficient
BS: Barkovich Score
M-GCN: Multi-scale Graph Convolution Network
PCA: Principal Component Analysis
GM: Gray Matter
